# Metabolic syndrome markers in wistar rats of different ages

**DOI:** 10.1186/1758-5996-4-16

**Published:** 2012-04-27

**Authors:** Ana Carolina Ghezzi, Lucieli Teresa Cambri, José Diego Botezelli, Carla Ribeiro, Rodrigo Augusto Dalia, Maria Alice Rostom de Mello

**Affiliations:** 1 Department of Physical Education, São Paulo State University (UNESP – Universidade Estadual Paulista), Rio Claro, SP, Brazil

**Keywords:** Metabolic syndrome, Biochemical parameters, Metabolism, Aging, Wistar rats

## Abstract

In recent decades, metabolic syndrome has become a public health problem throughout the world. Longitudinal studies in humans have several limitations due to the invasive nature of certain analyses and the size and randomness of the study populations. Thus, animal models that are able to mimic human physiological responses could aid in investigating metabolic disease. Thus, the present study was designed to analyze metabolic syndrome markers in albino Wistar rats (*Rattus norvegicus*) of different ages. The following parameters were assessed at two (young), four ( adult), six (adult), and twelve (mature) months of age: glucose tolerance (glucose tolerance test); insulin sensitivity (insulin tolerance test); fasting serum glucose, triglycerides, total cholesterol, HDL cholestero, and LDL cholesterol concentrations; glucose uptake in isolated soleus muscle; and total lipid concentration in subcutaneous, mesenteric, and retroperitoneal adipose tissue. We found that aging triggered signs of metabolic syndrome in Wistar rats. For example, mature rats showed a significant increase in body weight that was associated. In addition, mature rats showed an increase in the serum concentration of triglycerides, total cholesterol, and LDL cholesterol, which is characteristic of dyslipidemia. There was also an increase in serum glucose compared with the younger groups of animals. Therefore, aging Wistar rats appear to be an interesting model to study the changes related to metabolic syndrome.

## Introduction

Metabolic syndrome is characterized by a combination of risk factors for cardiovascular disease and diabetes that are generally linked to insulin resistance and central obesity [1]. Cardiovascular disease is the leading cause of morbidity and mortality in the world [2]; therefore, the study of metabolic syndrome as a predictor of cardiovascular problems is critical.

Aging correlates with an increase in the prevalence of cardiovascular disease, which is a health concern, especially because of increases in the life expectancy of the world population. The aging of the world population has caused concerns about the balance of the age pyramid. Studies have estimated that the proportion of people over 65 years of age, which was 5.2 % in 1950, will increase to 15.6 % in 2050; thus, the elderly will represent one fifth of the world population [3].

The prevalence of metabolic syndrome in the population of the United States is 20-25 % and close to 55 % for the elderly [4]. In Brazil, a metabolic syndrome prevalence of 52.3 % was observed in the elderly; however, due to different definitions for metabolic syndrome, the prevalence may differ among studies [3].

The most commonly used definition for metabolic syndrome is the one given by the World Health Organization (WHO), which requires the presence of diabetes or insulin resistance and two of the following characteristics: a high waist/hip ratio, a high concentration of triglycerides or a low concentration of HDL cholesterol, increased blood pressure and urinary excretion of albumin [5].

Research using animal models that develop a complete case of metabolic syndrome, including all of the above mentioned risk factors, are rare. Studies have demonstrated that the Wistar Ottawa Karlsburg (WOKW) line of rats develops a complete case of metabolic syndrome that includes obesity, moderate hypertension, dyslipidemia, glucose intolerance, and hyperinsulinemia [6].

Another circumstance that leads to the manifestation of metabolic syndrome symptoms in rodents is the aging process; however, manifestation differs between rat strains. For example, the Fisher 344 rat, which has been widely used in studies that separately assess metabolic syndrome characteristics [7], shows a small increase in body weight at 24 months of age. In this strain, adipocyte size is markedly increased, and plasma insulin concentrations are slightly higher in mature rats. Wistar rats at twenty-four months of age show similar blood insulin levels as adult rats at three months [8] despite the large increase in body weight and adipocyte hyperplasia [9]. Wistar rats also show a decrease in peripheral glucose uptake [10] as they age. Furthermore, recent studies have emphasized that the conditions considered as “standard” for the general maintenance of laboratory rodents (e.g., collective cages containing two to five animals per cage, with a flooring of wood shavings and free access to food) could favor the development of metabolic syndrome. This phenomenon could make rodent models suitable models of human obesity [11].

Relatively few studies have simultaneously monitored the development of various metabolic syndrome markers resulting from aging in Wistar rats, which have been widely used in experiments in Brazil and in several different studies conducted by our research group [12-14]. Therefore, the present study was designed to analyze metabolic syndrome markers in albino Wistar rats (*Rattus norvegicus*) at different ages.

## Materials and methods

### Animals

The present study used albino Wistar rats between two and twelve months of age. The rats were obtained from the central animal facility of São Paulo State University - UNESP – Botucatu, São Paulo, Brazil and were kept in the animal facility of the Department of Physical Education of the São Paulo State University – UNESP – Rio Claro, São Paulo, Brazil. The rats were fed a standard, balanced rodent diet LABINA (Purina®^,^ Composition: a control diet (57,3 % carbohydrate, 41,2 % of cornstach), 200 g protein, 70 g lipids, 50 g fiber, minerals and vitamins are balanced to the species) and water *ad libitum* and were kept in collective plastic cages (four per cage) at a room temperature of 25°C and a 12-h light/12-h dark cycle. The protocol for the animal studies was subjected to the Ethics Committee on Animal Use from the Institute of Biosciences, UNESP, Rio Claro (CEUA), under protocol 2638.

### Experimental design and groups

The rats were assessed for metabolic syndrome markers at different ages:

group 2 months (n = 8) – at two months (young adult);

group 4 months (n = 8) – at four months (adult);

group 6 months (n = 8) – at six months (adult);

group 12 months (n = 8) – at twelve months (mature).

### Assessment prior to sacrifice

· **General assessments:**All animals had their body weight, nasoanal length and food intake recorded each week. The food intake was calculated per cage, using the formula:

(1) $$\text{Food intake}=\frac{\text{Daily Food Intake}\left(\text{grams}\right)}{\sum \text{body weight of the rats in each cage}\left(\text{grams}\right)}$$

· **Oral glucose tolerance test – OGTT:**The glucose tolerance of the rats was assessed by the oral glucose tolerance test (OGTT). This test was performed during the last week of the experiment after 12 h of fasting and began when the first blood sample was taken from a cut at the tip of the tail (time 0). Subsequently, a 20 % glucose solution (2 g/kg body weight) was administered to the rats via a polyethylene gastric tube. Blood samples were collected after 30, 60, and 120 min with heparinized capillary tubes, and 25-μL aliquots were used to determine the glucose and insulin concentrations. A single cut at the tip of the tail was enough to collect all of the blood samples. Blood glucose concentrations were determined using the glucose-oxidase method, and insulin concentrations were determined using commercial enzyme-linked immunosorbent assay (ELISA) kits (LABORLAB®). The results were analyzed with ORIGIN software by calculating the area under the curve (AUC) for serum glucose and insulin using the trapezoidal method test [15].

· **Insulin tolerance test - ITT**Peripheral insulin sensitivity was assessed by the insulin tolerance test (ITT), which was carried out 48 h after the OGTT. After the ITT, a crystalline insulin solution (30 IU/100 g body weight, LILLY) was administered subcutaneously. Blood samples were collected after 30, 60, and 120 min with heparinized capillary tubes, and 25-μL aliquots were used to determine the concentration of glucose by the glucose-oxidase method. A single cut at the tip of the tail was enough to collect all of the blood samples. The glucose removal rate (KITT), which was expressed as %/minute, was calculated using the following formula: (0.0693/t/2) x 100. Using ORIGIN software, blood glucose (t/2) was calculated by the least squares curve analysis for blood glucose content from the moments when serum glucose decreased after insulin administration [16].

· **Animal sacrifice and collection of the biological material**When the rats reached a predetermined age, they were all sacrificed by decapitation under deep anesthesia with CO_2_ (i.e., 48 h after the last *in vivo* assessment). Blood samples were collected for serum separation for glucose, triglycerides, total cholesterol, LDL cholesterol, and HDL cholesterol concentrations determination by calorimetric methods using commercial kits LABORLAB).

· **Soleus muscle**Longitudinal muscle slices weighing approximately 25–35 mg were incubated in Krebs-Ringer bicarbonate buffer enriched with glucose (5.5 mM). The buffer contained [^3^ H]-2-deoxyglucose (2-DG, 0.5 μCi/mL) and insulin (100 mU/mL), and the slices were incubated in glass flasks for approximately 1.5 h under continuous gassing with carbogen (O_2_/CO_2_ ratio of 95 %/5 %) and constant stirring in a water bath (37°C). Glucose uptake was assessed using 2-DG as a marker and measuring the radioactivity of [^3^ H] using a beta counter.

· **Adipose tissue**The adipose tissue from the posterior subcutaneous, mesenteric and retroperitoneal were analyzed. Mesenteric and retroperitonial are visceral regions of the adipose tissue, they usually are used as markers of visceral adiposity.The excision of different fat deposits was performed according to methods described by Cinti [17]. Data were expressed as a relation between regional adipose tissue weight (grams)/body weight (100 grams).

### Statistics

The results were statistically analyzed using analysis of variance (ANOVA) with a pre-established significance level of 5 %. When needed, the Newman-Keuls post hoc test was used.

## Results

**Figure 1:**
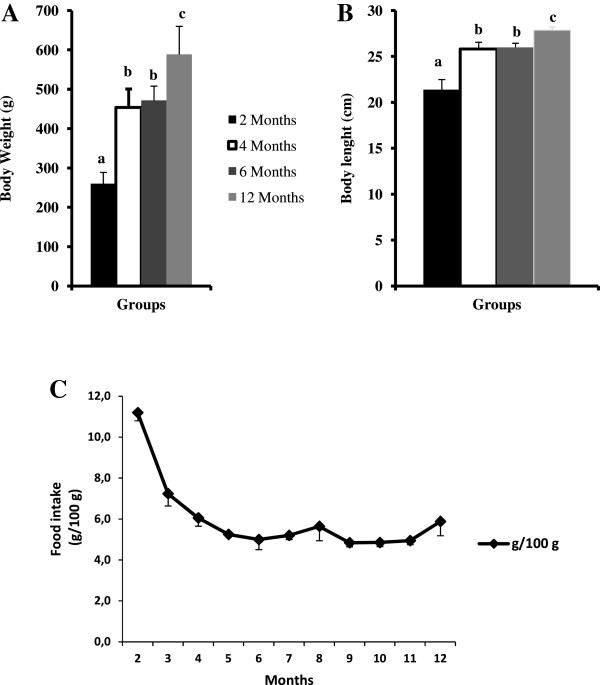
**The mean and standard deviation for body weight (A) and nasoanal length (B) from groups containing eight rats each.** All differences are expected in this range. Different letters mean different result. (One-way ANOVA and Newman-Keuls post hoc test, P < 0.05).

Figure 1 A, B and C show the increase in body weight, nasoanal length and food intake of the rats. Significant differences were observed in body weight and nasoanal length between two and four months of life. Additional significant increases in body weight and nasoanal length occurred from six to twelve months. For the figure we see that food intake to two months the animals eat more (g/100 g) and two months after intake decreases considerably, reaching an almost stable, with little fluctuation has one month to another.

**Figure 2:**
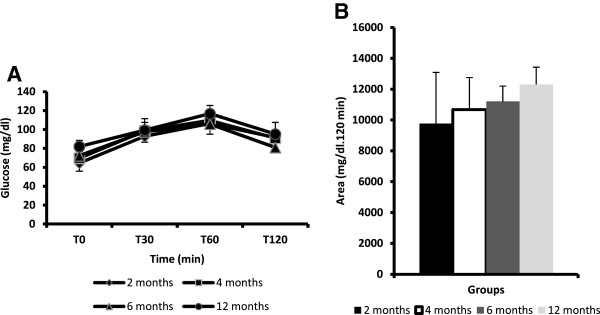
**The mean and standard deviation for the blood glucose values and the area under the curve of the blood glucose values during the oral glucose tolerance test (OGTT). Each group consisted of eight rats.** Note: There were no statistically significant differences between the groups (one-way ANOVA, P > 0.05).

Figure 2, A and B show values for the blood glucose before and 30, 60, and 120 min after the glucose load and for the area under the curve of the blood glucose during the oral glucose tolerance test (OGTT). There were no statistical differences between the groups.

**Figure 3:**
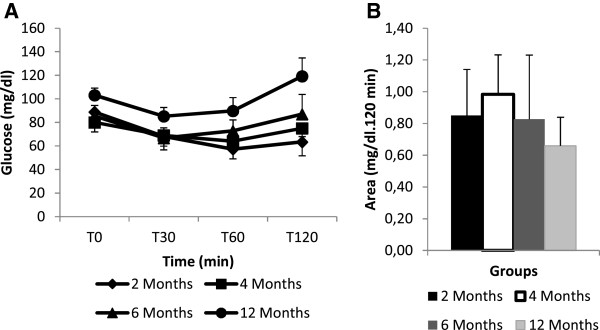
**The mean and standard deviation for the blood glucose and the glucose removal constant (KITT) values during the insulin sensitivity test (ITT). Each group consisted of eight rats.** Note: There were no statistically significant differences between the groups (one-way ANOVA, P > 0.05).

Figure 3, A and B show the blood glucose concentrations values before and 30, 60, and 120 min after exogenous insulin administration and the glucose removal constant (KITT) values during the insulin sensitivity test (ITT). There were no significant differences between the groups.

The values for the serum concentrations of glucose, triglycerides (mg/dL), total cholesterol, HDL cholesterol, and LDL cholesterol at the end of the experiment are shown in Table 1. Significant increases in serum glucose, triglycerides, total cholesterol, HDL cholesterol, and LDL cholesterol concentrations were observed in twelve-month-old rats compared with the other age groups.

**Table 1:** The means and standard deviations for the serum concentrations of glucose (mg/dL), triglycerides (mg/dL), total cholesterol (mg/dL), HDL cholesterol (mg/dL), and LDL cholesterol (mg/dL) (n = 8 per group)

**Age**	**Glucose**	**Triglycerides**	**Total Cholesterol**	**HDL**	**LDL**
2 months	98 ± 14^a^	105 ± 30^a^	82 ± 7^a^	29 ± 8^a^	64 ± 8^a^
4 months	109 ± 8^a^	100 ± 11^a^	100 ± 10^a^	48 ± 4^a^	67 ± 3^a^
6 months	119 ± 29^a^	125 ± 33^a^	100 ± 8^a^	40 ± 6^a^	63 ± 8^a^
12 months	152 ± 29^b^	260 ± 83^b^	171 ± 30^b^	79 ± 25^b^	121 ± 19^b^

Different letters indicate a statistically significant difference (one-way ANOVA and Newman-Keuls post hoc test, P < 0.05).

Table 2 shows the data for the total lipids from the mesenteric, retroperitoneal, and subcutaneous regions. A statistically significant difference was observed between groups for mesenteric and retroperitoneal regions. The two-month group had less fat accumulation in all regions than the other groups, whereas the twelve-month group had greater accumulation only in the mesenteric region.

**Table 2:** The means and standard deviations of total lipids (mg/100 g) in the adipose tissue from different regions (n = 8 per group)

**Groups**	**Mesenteric**	**Retroperitoneal**	**Subcutaneous**
2 months	1 005 ± 240^a^	770 ± 212^a^	692 ± 177^a^
4 months	2 267 ± 726^b^	2634 ± 536 ^b^	1 851 ± 403^b^
6 months	2475 ± 537^b^	2472 ± 408^b^	1 499 ± 381^b^
12 months	4399 ± 643^c^	3191 ± 703^b^	1 896 ± 758^b^

Different letters indicate a statistically significant difference (one-way ANOVA and Newman-Keuls post hoc test, P < 0.05).

**Figure 4:**
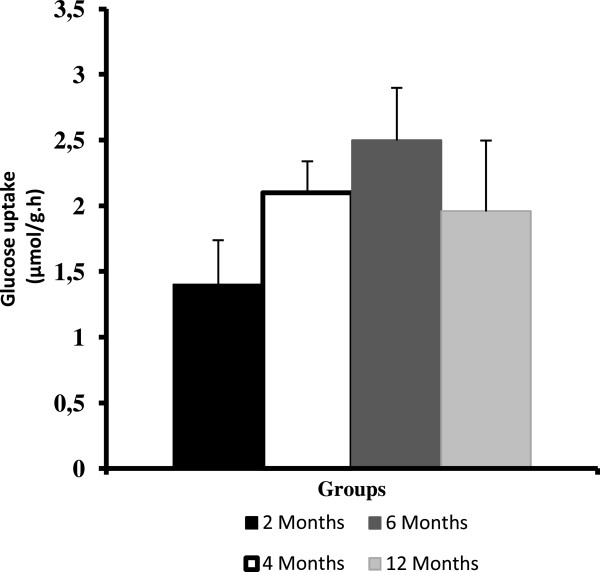
**The mean and standard deviation for the glucose uptake (μmol/g/h) by the isolated soleus muscle. Each group consisted of eight rats.** Note: There were no statistically significant differences between the groups (one-way ANOVA, P > 0.05).

Figure 4 presents the glucose uptake values for the isolated soleus muscle. There were no significant differences between the groups.

## Discussion

This study analyzed the effects of aging on the development of several metabolic syndrome markers in Wistar rats, which are often used in research, especially in Brazil.

The life expectancy of the world population has increased due to several factors, such as developments in medicine and improvements in basic sanitation. Longevity, however, is accompanied by several diseases. One condition that has attracted a lot of attention is metabolic syndrome because the prevalence of cases in the world population has increased exponentially [18]. Metabolic syndrome is association clinical signs of diseases that increase the risk of cardiovascular disease, which is the leading cause of death in the world.

Because of the relationship between metabolic syndrome and cardiovascular disease, there is a need to understand the mechanisms of metabolic syndrome. Thus, several studies have been conducted with experimental models because the analysis of metabolic syndrome mechanisms often includes invasive procedures, which are restricted from being used in humans. Magnetic resonance spectroscopy in humans provides a non invasive way to measure fat, glucose and glycogen in humans and rodents, and many such studies show such longitudinal and dynamic measures can successfully be made [19].Rats and mice are among the animals most commonly used to study metabolic syndrome, and several rodent strains have been used.

The present study used albino Wistar rats and found that the rats in the two-month group had lower body weights compared with the other groups. The rats shows a marked gain in body weight from two to four months due to their stage of growth and development. Although body weight stabilized from four to six months, there was another significant weight increase at twelve months. A similar result has also been observed by Lerco et al. [20] a in Wistar rats. The food intake profile data showed that after two months there is stabilization of food intake, which agrees with literature data [21,22]. Similar to human beings, rats ate more during accelerated body growth with subsequent stabilization. These data indicate that increasing the weight gain of animals and an increase of mesenteric fat is not related to the increased food intake.

Ford et al. [4] used criteria defined by the NCEP and found that the prevalence of both obesity and metabolic syndrome in humans increases with age.

The results of the OGTT and ITT at two, four, six, and twelve months of age demonstrated that there was no change in the blood glucose level throughout the experimental period (i.e., neither glucose tolerance nor insulin sensitivity were affected). These findings were in agreement with previous studies that reported that Wistar rats at twenty-four months of age have similar blood insulin and blood glucose levels as three-month-old adult rats [8] despite increases in body weight and adipocyte hypertrofy [9].

Metabolic syndrome comprises several physiological disorders and those changes also affect the glucose transporters, regulation of homeostasis intra and extra-cellular glucose is directly related to controlling the expression of genes that encode different glucose transporters [23]. The stimulation of insulin causes the translocation of GLUT4 to happen toward the plasma membrane thereby increasing glucose uptake, participating significantly in the control of glucose homeostasis [24].

At twelve months, the blood biochemistry of the rats showed an increase in the levels of triglycerides, total cholesterol, and LDL cholesterol, which was characteristic of the onset of dyslipidemia. Interestingly, Mota et al. reported different results [12]; however, their animals were assessed for a shorter period of time (i.e., from weaning to 120 days of age), which could explain the difference between the present study and the study by Mota et al. [12]. The maintenance of high-circulating triglycerides concentration causes an imbalance in lipid and carbohydrate oxidation [25]. This phenomenon can increase plasma glucose, as observed in the present study was an increase in blood glucose at twelve months compared with the younger age groups. Interestingly, studies of mature diabetic patients have shown that these individuals have a higher risk to develop dyslipidemia than nondiabetic individuals [26].

Another factor that has been associated with metabolic syndrome is visceral fat. Fat distribution is reported to be more closely linked to metabolic disorders than total body weight, and fat distribution is even more significant in older individuals [27] Therefore, central body fat is a more reliable factor in the diagnosis of metabolic syndrome and other morbidities than the classification of the degree of obesity. Importantly, visceral fat is closely linked with the development of insulin resistance and other glucose alterations disorders.

Because glucose is an important substrate for muscles, it is of value to investigate the muscle metabolism of this substrate during the aging process in Wistar rats. Thus, the present study assessed glucose uptake in incubated soleus muscle. Interestingly, there were no statistically significant differences between the groups. These data can reveal the cause-effect order in the metabolic syndrome markers.

As described before, the lipid metabolism was altered; this change can lead to future alterations in the carbohydrate metabolism [28]. The twelve month group showed higher concentration of serum glucose compared to the younger. This can be the first metabolic alteration found. Studies from our laboratory [29,30] using fructose to induce metabolic syndrome in rats, have demonstrated that the alterations in the lipid metabolism are more evident than glucose alterations.

Studies have shown that aging causes the spontaneous development of obesity and insulin resistance in rats. In addition, some studies have reported changes in glucose transporter type 4 (GLUT 4) content in mature rats; however, these studies used different rat strains and analyzed different muscles [31].

## Conclusion

The aging process triggered metabolic syndrome characteristics in Wistar rats. Mature rats showed a significant increase in body weight and lipid content in adipose tissue. In addition, mature rats showed characteristics of dyslipidemia, such as increased serum triglycerides, total cholesterol, and LDL cholesterol. Similarly, mature rats showed an increase in serum glucose compared with younger groups. Therefore, aging Wistar rats appear to be an interesting model for the study of the changes related to metabolic syndrome.

## Competing interest

The authors have no conflicts of interest, and no section of the article has been or will be published in another journal.

## Authors’ contributions

ACG was responsible for experimental design, data collection, statistical analysis and preparation of the manuscript. LTC was responsible for experimental design, data collection and preparation of the manuscript. JDB and RAD was responsible for collecting data and preparing the manuscript. MARM, NO ARMM was responsible for experimental design and coordination of research. All authors read and approved the final manuscript.

## References

[B1] Duncan BB Schimidt MA Chronic activation of the innate immune system may underlie the metabolic syndrome Sao Paulo Med J 2001 119 122 127 10.1590/S1516-31802001000300008 11391456 PMC11164471

[B2] World Health Organization (WHO) Preventing chronic diseases: a vital investment 2005

[B3] Brazilian Institute of Geography and Statistics (IBGE - Instituto Brasileiro de Geografia e Estatística) Profile of Mature Heads of Households (Perfil dos Idosos Responsáveis pelos Domicílios) 2002 http://www.ibge.gov.br/home/presidencia/noticias/25072002pidoso.shtm

[B4] Ford ES Giles WH Dietz WH Prevalence of the metabolic syndrome among US adults: findings from the Third National Health and Nutrition Examination Survey JAMA 2002 287 356 359 10.1001/jama.287.3.356 11790215

[B5] WHO Third International Conference on Health Promotion 1991 Sweden: Sundsvall http://www.who.int/hpr/NPH/docs/sundsvall_statement.pdf Accessed on 06 April 2009]. Available from:

[B6] Van den Brandt J Kovacs P Kloting I Metabolic features in disease-resistant as well as in hypertensive SHR and newly established obese WOKW inbred rats Int J Obes Relat Metab Disord 2000 24 1618 1622 10.1038/sj.ijo.0801444 11126214

[B7] Cartee GD Briggs-Tung C Kietzke EW Persistent effects of exercise on skeletal muscle glucose transport across the life-span of rats J Appl Physiol 1993 75 972 978 8226503 10.1152/jappl.1993.75.2.972

[B8] Carrascosa JM Ruiz P Martinez C Pulido JA Satru SJ Andres A Insulin receptor kinase activity in rat adipocytes is decreased during aging Biochem Biophys Res Commun 1989 160 303 309 10.1016/0006-291X(89)91656-2 2653319

[B9] Newby FD Digirolamo M Cotsonis GA Kutner MH Model of spontaneous obesity in aging male Wistar rats Am J Physiol Regul Integr Comp Physiol 1990 259 1117 1125 10.1152/ajpregu.1990.259.6.R1117 2260722

[B10] Escriva E Agote M Rubio EJ Molero JCA Pascual-Leone AM Andres SAJ In Vivo Insulin-Dependent Glucose Uptake of Specific Tissues Is Decreased during Aging of Mature Wistar Rats Endocrinology 1997 138 49 54 10.1210/en.138.1.49 8977384

[B11] Martin B Ji S Maudsley S Mattson MP “Control” laboratory rodents are metabolically morbid: Why it matters Proc Natl Acad Sci 2010 107 E134 E147 10.1073/pnas.1008118107 20194732 PMC2852022

[B12] Mota CSA Ribeiro C Araujo GG Araujo MB Manchado FB Oliveira CAM Exercise training in the aerobic/anaerobic metabolic transition prevents glucose intolerance in alloxan-treated rats BMC Endocr Disord (Online) 2008 8 11 10.1186/1472-6823-8-11 PMC2567313 18828926

[B13] Ghezzi AC Cambri LT Ribeiro C Botezelli JD Mello MAR Impact of early fructose intake on metabolic profile and aerobic capacity of rats Lipids in Health Disease 2011 10 3 10.1186/1476-511X-10-3 PMC3024237 21223589

[B14] Cambri LT Ghezzi AC Ribeiro C Dalia RA Mello MAR Recovery of rat growth and lipid profiles in adult rats subjected to fetal protein malnutrition with a fructose-rich diet Nutr Res 2010 30 156 162 10.1016/j.nutres.2010.01.001 20227002

[B15] Mathews JNS Altaman DG Campbell MJ Royston P Analysis of serial measurements in medical research Br Med J 1990 27 230 235 10.1136/bmj.300.6719.230 PMC1662068 2106931

[B16] Bonora E Moghetti P Zancanaro C Cigolini M Querena M Cacciatori V Estimates of in vivo insulin action in man: Comparison of insulin tolerance tests with euglycemic and hyperglycemic glucose clamp studies J Clin Endocrinol Metab 1989 68 374 378 10.1210/jcem-68-2-374 2645308

[B17] Cinti S The adipose organ Prostagl Leukotr Essent Fatty Acids 2005 73 9 15 10.1016/j.plefa.2005.04.010 15936182

[B18] Meigs JB Epidemiology of the metabolic syndrome Am J Manag Care 2002 8 11 suppl S283 S292 12240700

[B19] Boesch C Décombaz J Slotboom J Kreis R Observation of intramyocellular lipids by means of 1H magnetic resonance Spectroscopy Proc Nutr Soc 999 58 841 850 10817151 10.1017/s0029665199001147

[B20] Lerco MM Spadella CT Machado JLM Schellini AS Padovani CR Characterization of an experimental model of Diabetes Mellitus induced by alloxan in rats. Clinical and laboratory study Acta Cir Bras 2003 18 132 142

[B21] Takano S Kanai S Hosoya H Ohta M Uematsu H Miyasaka K Orexin-A does not stimulate food intake in old rats AJP - GI 2004 287 81 88 10.1152/ajpgi.00218.2004 15271651

[B22] Morley JE Decreased Food Intake With Aging J Gerontol A Biol Sci Med Sci 2001 56 81 88 10.1093/gerona/56.suppl_2.81 11730241

[B23] Stenbit AE Tsao Tsu-Shuen Li J Burcelin R Geenen DL Factor SM Houseknecht K Katz EB Charron MJ GLUT4 heterozygous knockout mice develop muscle insulin resistance and diabetes Nat Med 1997 3 1096 1101 10.1038/nm1097-1096 9334720

[B24] Rea S James DE Moving GLUT4: The biogenesis and trafficking of GLUT4 storage vesicles Diabetes 1997 46 1667 1677 10.2337/diabetes.46.11.1667 9356011

[B25] Brown MS Goldstein JL The SREBP pathway: regulation of cholesterol metabolism by proteolysis of a membranebound transcription factor Cell 1997 89 331 340 10.1016/S0092-8674(00)80213-5 9150132

[B26] Garvey WT Kwon S Zheng D Shaughnessy S Wallace P Hutto A Pugh K Jenkins AJ Klein RL Liao Y Effects of insulin resistance and type 2 diabetes on lipoprotein subclass particle size and concentration determined by nuclear magnetic resonance Diabetes 2003 52 453 462 12540621 10.2337/diabetes.52.2.453

[B27] Forouhi NG Sattar N McKeigue PM Relation of C-reactive protein to body fat distribution and features of the metabolic syndrome in Europeans and South Asians Int J Obes Relat Metabol Disord 2001 25 9 1327 1331 10.1038/sj.ijo.0801723 11571595

[B28] Machado UF Shimizu I Saito M Reduced content and preserved translocation of glucose transporter (GLUT 4) in white adipose tissue of obese mice Physiol Behav 1994 55 621 625 10.1016/0031-9384(94)90035-3 8190786

[B29] Botezelli JD Dalia RA Reis IMR Barbieri RA Rezende TM Pelarigo JG Codogno J Gonçalves R Mello MAR Chronic consumption of fructose rich soft drinks alters tissue lipids of rats Diabetol Metabol Syndr 2010 2 43 10.1186/1758-5996-2-43 PMC2913938 20573247

[B30] Botezelli JD Mora RF Dalia RA Moura LP Cambri LT Ghezzi AC Voltarelli FA Mello MA Exercise counteracts fatty liver disease in rats fed a fructose-rich diet Lipids Health Dis 2010 9 116 10.1186/1476-511X-9-116 20946638 PMC2964725

[B31] Gulve EA Henriksen EJ Rodnick KJ Youn JH Holloszy JO Glucose transporters and glucose transport in skeletal muscles of 1- to 25-mo-old rats Am J Physiol 1993 264 3Pt1 E319 E327 8460679 10.1152/ajpendo.1993.264.3.E319

